# Supplementary material for: Identification and functional analysis of non-coding regulatory small RNA FenSr3 in Bacillus amyloliquefaciens LPB-18
Source: PeerJ. 2023 May 15;11:e15236. doi: 10.7717/peerj.15236 (PMC10194069; doi:10.7717/peerj.15236)
Supplement: Supplemental Information 4 [file peerj-11-15236-s004.zip › KO/CK-vs-T1_map/map04713.html]

KEGG PATHWAY: Circadian entrainment - Reference pathway


|  |  |
| --- | --- |
| **Circadian entrainment - Reference pathway** |  |

[
Pathway menu
| Organism menu
| Pathway entry
| Show description
| User data mapping
]

Circadian entrainment is a fundamental property by which the period of the internal biological clock is entrained by recurring exogenous signals, such that the organism's endocrine and behavioral rhythms are synchronized to environmental cues. In mammals, a master clock is located in the suprachiasmatic nuclei (SCN) of the hypothalamus and may synchronize circadian oscillators in peripheral tissues. Light signal is the dominant synchronizer for master SCN clock. Downstream from the retina, glutamate and PACAP are released and trigger the activation of signal transduction cascades, including CamKII and nNOS activity, cAMP- and cGMP-dependent protein kinases, and mitogen-activated protein kinase (MAPK). Of non-photic entrainment, important phase shifting capabilities have been found for melatonin, which inhibits light-induced phase shifts through inhibition of adenylate cyclase (AC). Multiple entrainment pathways converge into CREB regulation. In turn, phosphorylated CREB activates clock gene expression.

|  |  |  |
| --- | --- | --- |
| Reference pathway | 184% 150% 122% 100% 82% 67% 55% | 图片下载 |
